# New Evidence for the Role of Pituitary Adenylate Cyclase-Activating Polypeptide as an Antimicrobial Peptide in Teleost Fish

**DOI:** 10.3390/antibiotics12101484

**Published:** 2023-09-27

**Authors:** Janet Velázquez, Tania Rodríguez-Cornejo, Tania Rodríguez-Ramos, Geysi Pérez-Rodríguez, Laura Rivera, James Hugh Campbell, Lowia Al-Hussinee, Yamila Carpio, Mario Pablo Estrada, Brian Dixon

**Affiliations:** 1 Animal Biotechnology Department, Center for Genetic Engineering and Biotechnology (CIGB), P.O. Box 6162, Havana 10600, Cuba; janet.velazquez@cigb.edu.cu (J.V.); gey9412@gmail.com (G.P.-R.); 2 Department of Biology, University of Waterloo, 200 University Ave W., Waterloo, ON N2L 3G1, Canada; tania.rodriguez@upch.pe (T.R.-C.); tania.rodriguez-ramos@uwaterloo.ca (T.R.-R.); l2rivera@uwaterloo.ca (L.R.); jhcampbell@uwaterloo.ca (J.H.C.); jlowia@gmail.com (L.A.-H.)

**Keywords:** antibacterial activity, PACAP, rainbow trout, RTS11, *Yersinia ruckeri*

## Abstract

Pituitary Adenylate Cyclase-Activating Polypeptide (PACAP) is a multifunctional neuropeptide that is widely distributed and conserved across species. We have previously shown that in teleost fish, PACAP not only possesses direct antimicrobial properties but also immunomodulatory effects against the bacterial pathogens *Flavobacterium psychrophilum* and *Pseudomonas aeruginosa* using in vitro and in vivo experiments. These previous results suggest PACAP can be used as an alternative to antibiotics to prevent and/or treat bacterial infections in the aquaculture industry. To accomplish this goal, more studies are needed to better understand the effect of PACAP on pathogens affecting fish in live infections. In the present study, the transcripts PACAP, PRP/PACAP, and VPAC2 receptor were examined in rainbow trout (*Oncorhynchus mykiss*) naturally infected with *Yersinia ruckeri*, which exhibited an increase in their expression in the spleen when compared to healthy fish. Synthetic *Clarias gariepinus* PACAP-38 has direct antimicrobial activity on *Y. ruckeri* and inhibits up to 60% of the bacterial growth when the peptide is at concentrations between 50 and 100 µM in TSB. The growth inhibition increased up to 90% in the presence of 12.5 µM of PACAP-38 when salt-free LB broth was used instead of TSB. It was also found to inhibit *Y. ruckeri* growth in a dose-dependent manner when the rainbow trout monocyte/macrophage-like cell line (RTS11) was pre-treated with lower concentrations of the peptide (0.02 and 0.1 µM) before going through infection. Differential gene expression was analyzed in this in vitro model. Overall, the results revealed new evidence to support the role of PACAP as an antimicrobial and immunomodulatory peptide treatment in teleosts.

## 1. Introduction

In the last few years, neuropeptides have been shown to play a role in the host defense, including immunomodulatory action and direct antimicrobial properties, in addition to their conventional role as neurotransmitters [1]. Pituitary adenylate cyclase-activating polypeptide (PACAP) is part of the secretin/growth hormone-releasing hormone (GHRH)/vasoactive intestinal peptide (VIP) family [2]. PACAP splicing variants (forms with 38 or 27 amino acids) are broadly distributed in the nervous system and in peripheral organs [3]. Because of its remarkably conserved functions among vertebrates and its biomedical interest, the neuropeptide PACAP has been widely studied. PACAP-38 shares characteristics with canonical antimicrobial peptides (AMPs), including a basic charge of +10 at neutral pH and a structurally amphipathic arrangement with a notable complement of hydrophobic residues. It also shows strong antimicrobial activity similar in mechanical action on bacterial membranes to that of synthetic and natural AMPs. PACAP-38 and analogs have a strong antimicrobial response against gram-negative bacteria such as *Escherichia coli* and *Pseudomonas aeruginosa* and the gram-positive bacteria *Staphylococcus aureus* [2].

In teleost fish, multiple studies have focused on the effects of PACAP-38 on growth, food intake, immunomodulatory properties, and direct/indirect antimicrobial activity, among others. The genes encoding the two PACAP splicing variants and their receptors are modulated in the kidney and spleen during viral (Viral Haemorrhagic Septicaemia virus—VHSV) and bacterial (*Yersinia ruckeri*) septicaemic infections in brown trout [4]. It has also been demonstrated that *Clarias gariepinus* PACAP-38 has direct antimicrobial activity against bacteria of concern in aquaculture and human health [5,6]. In vitro studies conducted in a monocyte/macrophage-like rainbow trout cell line (RTS11) first demonstrated evidence of the antimicrobial activity of PACAP-38 during an in vitro live infection model with the aquatic pathogen *Flavobacterium psychrophilum*. In addition, PACAP-38 exerts immunostimulatory activity on rainbow trout immune cells [6]. We reported the first evidence of PACAP-38 antiviral activity in rainbow trout fry experimentally infected with VHSV, with an increased survival of PACAP-treated fish in immersion baths [7]. More recently, the antibacterial and immunostimulatory activity of PACAP-38 against *P. aeruginosa* in *C. gariepinus* by an in vivo injection/challenge experiment provided the first evidence of a link between PACAP and the expression of the antimicrobial peptides Hepcidin and Pardaxin [8].

*Yersinia ruckeri* is a facultative, gram-negative rod with rounded ends, belonging to the Enterobacteriaceae family [9]. This bacterium constitutes the etiological agent of Enteric Redmouth Disease (ERM) or Yersiniosis, and it’s one of the main bacterial diseases in salmonids [10]. Although this bacterial infection affects different teleost species, rainbow trout are the most susceptible [11,12]. This disease affects fish at all stages, but it is usually acute in fingerlings and chronic in adult fish [13]. Mortality after acute infections varies between 30 and 70% [14,15]. Different strategies have also been conducted to date to control this disease, such as: (a) vaccines (injection and immersion vaccination using a bacterin of formalin-killed *Y. ruckeri* serotype O1) [16,17,18,19,20], (b) immunostimulants (β-1,3-glucan from *Euglena gracilis* [21], *Coriandrum sativum* extract [22] and aqueous methanolic extract of thin-skinned plum (*Prunus domestica*) [23]), (c) prebiotics (non-digestible oligosaccharides, lactulose, lactitol and inulin [24]), (d) probiotics (*Carnobacterium maltaromaticum* B26, *Carnobacterium divergens* B33 [25] and *Lactobacillus plantarum* 426951 [26]) and even lately the use of (e) beneficial postbiotics (antimicrobial compounds after microbial inactivation and end products of bacterial growth in the absence of viable cells [27]). All these approaches conferred some protective effects against ERM [28]. Nevertheless, the treatment of the disease is usually based on the administration of different antibiotics, such as amoxicillin, oxolinic acid, oxytetracycline, and sulfadiazine, in combination with trimethoprim and florfenicol [29]. 

In the context of the rapid emergence of different antimicrobial-resistant bacterial strains, the use of AMPs represents an attractive alternative to classical antibiotics. So far, AMP application for the control and treatment of Yersiniosis in rainbow trout has only been investigated previously by Chettri et al. [30], but the authors concluded that in-feed oral administration of the AMP CAP18 was unsuccessful in controlling the disease. In this context, the present work aimed to further characterize *C. gariepinus* PACAP-38’s function as an effective antimicrobial and immunostimulatory agent against *Y. ruckeri*, a prominent bacterial pathogen in rainbow trout aquaculture. With this purpose in mind, we first evaluated the expression of cDNA encoding PACAP splicing variants (PACAP and PACAP Related Peptide (PRP/PACAP)) and the VPAC2 receptor in naturally infected rainbow trout with *Y. ruckeri*. We also tested the direct and indirect antimicrobial activity of PACAP-38 on *Y. ruckeri*. Overall, the results confirmed PACAP-38 has antimicrobial and immunomodulatory activities in teleost fishes. 

## 2. Results

### 2.1. Up-Regulation of PACAP Splicing Variants and VPAC2 in the Spleen of Yersinia ruckeri-Infected Trout

**Figure 1:**
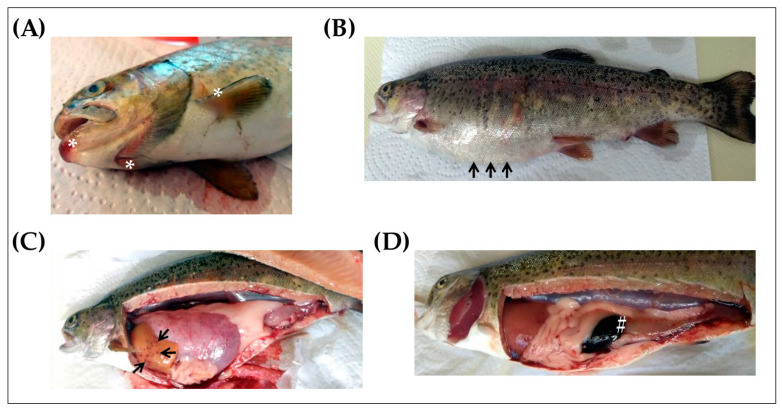
Clinical signs of *Yersinia ruckeri* infection in rainbow trout (*Oncorhynchus mykiss*). Presence of subcutaneous hemorrhages in the oral cavity (**A**, white asterisks) and abdominal distension (**B**, black arrows). Internal lesions related to Yersiniosis included the presence of petechiae in the liver (**C**, black arrows) and enlarged spleen (**D**, white # symbol).

Before determining the effects that Yersiniosis had on the expression levels of cDNA encoding rainbow trout PACAP splicing variants (PACAP and PRP/PACAP) and VIP/PACAP receptor subtype 2 (VPAC2) in the spleen, head, kidney, and skin of rainbow trout, the presence of the disease was determined by visual examination, microbiological testing, and conventional PCR. We first searched for clinical signs suggestive of ERM (lethargy, swimming near the surface, exophthalmia, melanosis, distended abdomen, and subcutaneous hemorrhages in the oral cavity and base of fins), and fish that presented some of the related symptoms were selected for a detailed examination. As shown in Figure 1, almost all infected fish presented abdominal distension (Figure 1A), subcutaneous hemorrhages in the oral cavity and base of fins (Figure 1B), petechiae in internal organs such as the liver (Figure 1C), and enlarged and darkened spleen and intestinal inflammation (Figure 1D), confirming the presence of the disease. 

**Figure 2:**
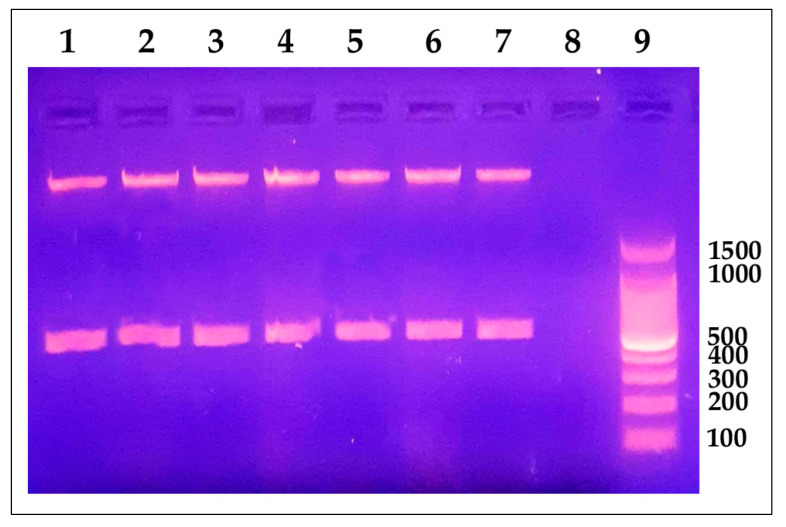
*Yersinia ruckeri* molecular identification using conventional PCR. Agarose gel electrophoresis 1% (p/v) showed an amplification product of 575 bp corresponding to a fragment of the 16S rRNA gene. Lane 1: positive control (*Y. ruckeri* ATTC^®^ 29473). Lanes 2 to 7: representative positive samples. Lane 8: PCR-negative control (DNase-free water). Lane 9: 100 bp DNA Ladder (Promega, Madison, WI, USA).

We also performed molecular detection of the pathogen using the presence of the *Y. ruckeri* 16S rRNA gene in tested samples by conventional and quantitative PCR (qRT-PCR). All samples from infected fish were positive for the gene of interest. We observed a single band with an electrophoretic migration according to the expected size of 575 bp (Figure 2). We also confirmed the presence of the pathogen by qRT-PCR analysis, where fish samples with CT values of 30.00 or less were considered infected and included in the infected group. Afterwards, we obtained a mean CT value of 32.26 ± 1.16 in control fish, while infected fish had a mean CT value of 16.44 ± 3.70 (Supplementary Table S1). 

**Figure 3:**
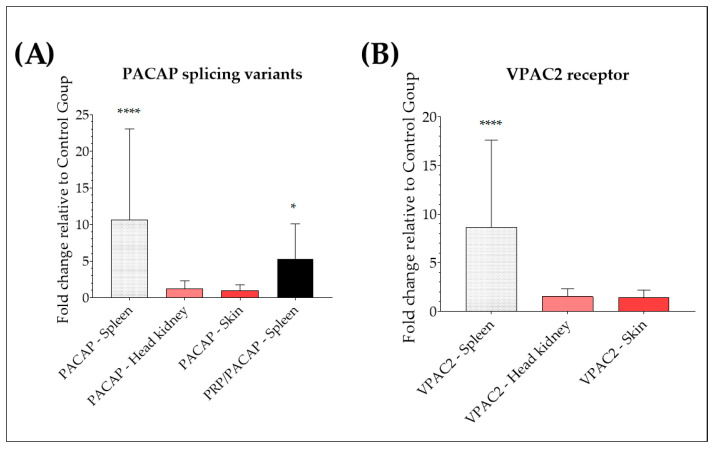
Analysis of PACAP splicing variants and VPAC2 genes in rainbow trout tissues after natural infection with *Yersinia ruckeri*. Relative expression of PACAP and PRP/PACAP genes (**A**) and VPAC2 receptor (**B**) in the spleen, head, kidney, and skin of *Y. ruckeri* naturally infected trout and healthy control trout. Relative expression was determined following the 2^−ΔΔCT^ method, and EF-1α was used as the reference gene. The data is expressed as a fold change relative to the expression level in the control group. Values are shown as the mean ± SD (N = minimum of 21 infected and a minimum of 17 healthy trout). Differences in fold change between groups were analyzed by a Mann–Whitney U test, * (*p* < 0.05) and **** (*p* < 0.0001).

Relative expression of cDNA encoding for rainbow trout PACAP, PRP/PACAP, and VPAC2 was assessed in *Y. ruckeri*-infected fish and compared with healthy control fish by qRT-PCR. An up-regulation of PACAP was observed in the spleen of naturally infected trout, with a 15-fold increase relative to the expression level in healthy trout (*p* < 0.0001) (Figure 3A). No significant differences were found in the head, kidney, or skin (*p* > 0.05, respectively). For the gene encoding PRP/PACAP, as shown in Figure 3A, a significant up-regulation was seen in the spleen, with an increase of 2-fold (*p* < 0.05). However, no expression of the gene of interest was detected in the head, kidney, or skin of naturally infected fish or healthy controls. Also, the natural infection with *Y. ruckeri* provoked a significant up-regulation of the VPAC2 receptor in the spleen, with a 5-fold increase in naturally infected trout relative to the expression level in healthy trout (*p* < 0.0001) (Figure 3B). No significant differences in VPAC2 mRNA levels were found in the head, kidney, and skin (*p* > 0.05, respectively).

### 2.2. Direct Antimicrobial Activity of Synthetic PACAP-38 against Yersinia ruckeri in Tryptic Soy Broth

**Figure 4:**
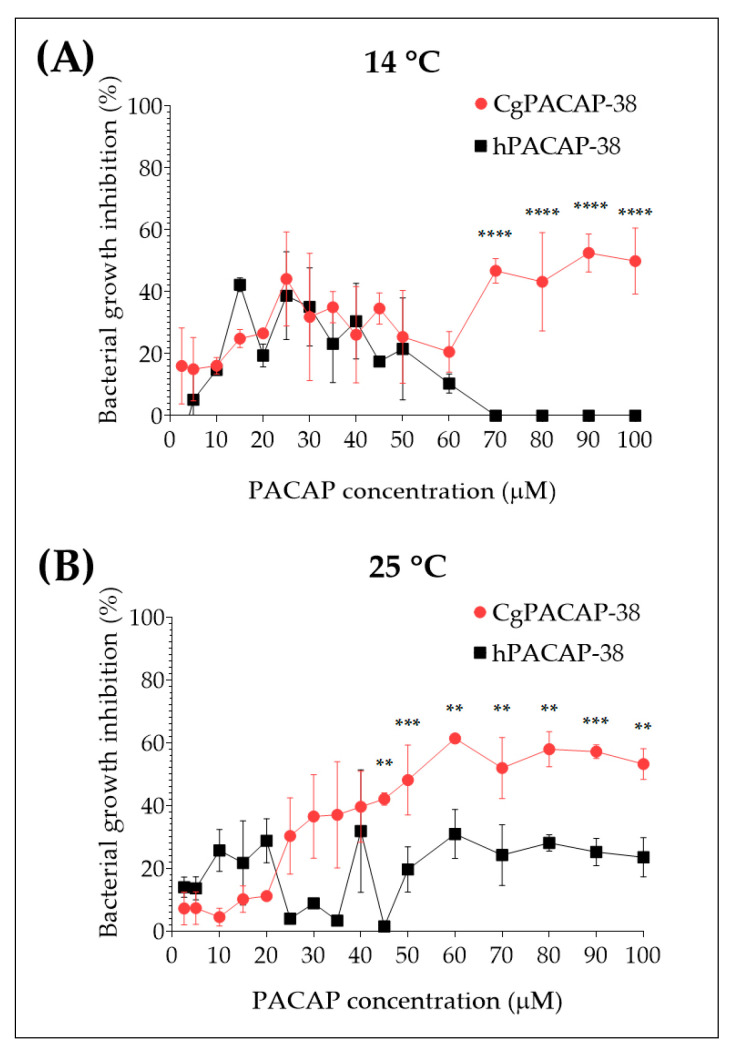
Direct antimicrobial activity of synthetic *Clarias gariepinus* PACAP-38 and human PACAP-38 against a *Y. ruckeri* isolate by broth microdilution peptide assay. Direct microbial activity of both PACAP-38 versions was determined at 14 °C (**A**) and 25 °C (**B**) against *Y. ruckeri* at increasing concentrations from 2.5 to 100 μM. Values are shown as the mean ± SD (N = 3). Differences among treatments were considered to be significantly different by a two-way ANOVA followed by Sidak’s multiple comparisons post-hoc test, ** (*p* < 0.01), *** (*p* < 0.001), and **** (*p* < 0.0001).

The direct antimicrobial activity of synthetic *Clarias gariepinus* PACAP-38 (CgPACAP-38) and human PACAP-38 (hPACAP-38) against *Y. ruckeri* was assessed by a broth microdilution peptide assay in Tryptic Soy Broth (TSB). We tested the minimal inhibitory concentrations of both PACAP-38 versions at 14 and 25 °C (Figure 4). We did not observe an in vitro bacterial growth inhibition of *Y. ruckeri*, when PACAP concentrations ranged from 2.5 to 50 μM, were used. At 14 °C, only CgPACAP-38 showed the best performance, inhibiting bacterial growth of *Y. ruckeri* by up to ~60% at concentrations of 70 µM or higher (*p* < 0.0001) (Figure 4A). On the contrary, hPACAP-38 displayed a negative trend in bacterial growth inhibition at 14 °C, with ~20% at 50 µM and no growth inhibition at 100 µM. In the case of the analysis performed at 25 °C, only CgPACAP-38 showed to be effective by inhibiting ~60% of *Y. ruckeri* bacterial growth from 45 to 100 µM (*p* < 0.01), while hPACAP-38 only inhibits ~20% of the bacterial growth (Figure 4B). In summary, these results indicated that only high concentrations of CgPACAP-38 can inhibit the in vitro growth of *Y. ruckeri* in TSB, with a Pearson r correlation of r = 0.7854 at 14 °C (*p* < 0.001) and r = 0.8993 at 25 °C (*p* < 0.0001). This effect appeared to be temperature-dependent. No correlation was observed for hPACAP-38 (*p* > 0.05). 

### 2.3. Effect of Different Salt Concentrations in Luria-Bertani Media on the Direct Antimicrobial Activity of Synthetic PACAP-38 

**Figure 5:**
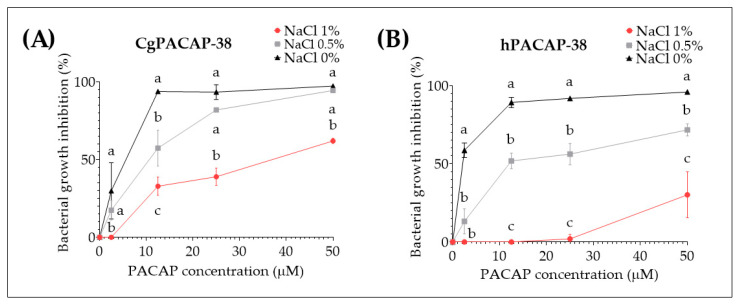
Direct antimicrobial activity of synthetic *Clarias gariepinus* PACAP-38 (**A**) and human PACAP-38 (**B**) against a *Y. ruckeri* isolate by broth microdilution peptide assay in Luria-Bertani broth. Direct microbial activity of both PACAP-38 versions was determined at 14 °C against *Y. ruckeri* at increasing concentrations from 0 to 50 μM with three different NaCl salt concentrations (0, 0.5, or 1%). Values are shown as the mean ± SD (N = 3). Differences among treatments were considered to be significantly different by a two-way ANOVA followed by Tukey’s multiple comparisons post-hoc test. Different lowercase letters represent statistically significant differences at *p* < 0.05. The significance of the letters is indicated in Supplementary Tables S2 and S3.

Taking into account the previous in vitro results where no significant impact of PACAP-38 on *Y. ruckeri* growth at concentrations ranging from 2.5 to 50 μM was observed in TSB, we decided to test the effect of different salt concentrations and a different media (Luria-Bertani media (LB)) on PACAP direct antibacterial activity. After 24 h incubation at 14 °C with 1%, 0.50%, or 0% NaCl, we observed that CgPACAP-38 was able to inhibit the bacterial growth (~93 to 97%) in the complete absence of salt from 12.5 to 50 μM and displayed a significantly different antibacterial activity among all salt concentrations at 12.5 μM (*p* < 0.01) (Figure 5A). Also, hPACAP-38 was able to inhibit bacterial growth (~89 to 96%) with 0% NaCl from 2.5 to 50 μM, while higher NaCl concentrations resulted in a significantly decreased antibacterial activity of hPACAP-38 (*p* < 0.001) (Figure 5B).

### 2.4. Effect of PACAP-38 on Yersinia ruckeri Bacterial Growth in Infected RTS11 

**Figure 6:**
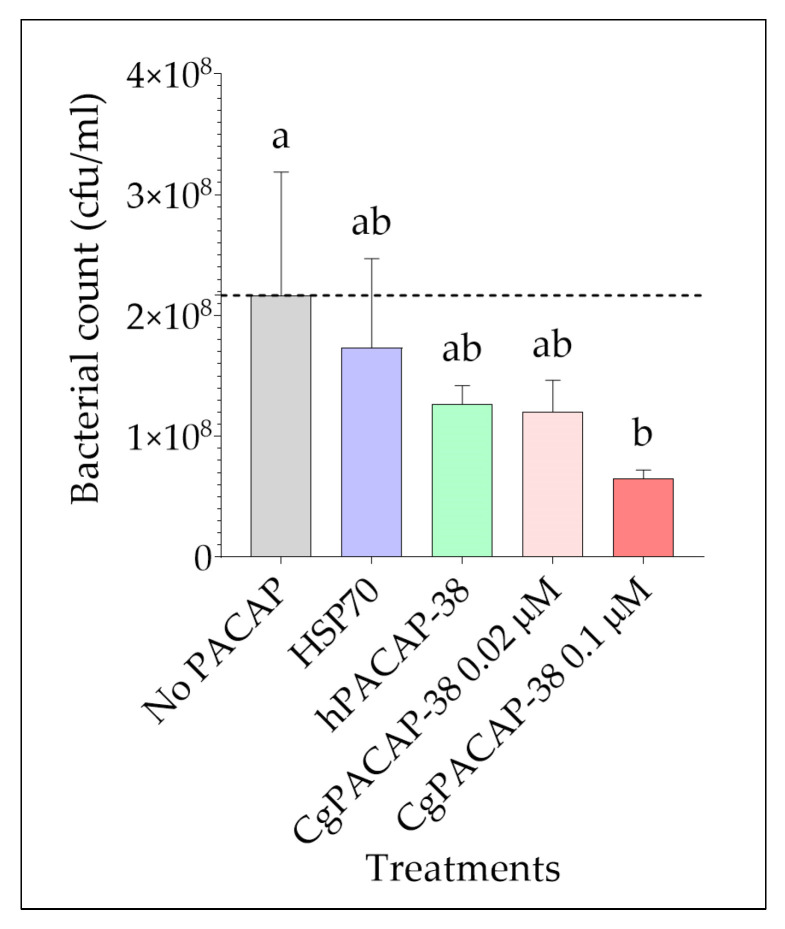
Quantification of *Yersinia ruckeri* by standard plate count (SPC) of cell culture media during live infection (MOI of 0.001) of PACAP-treated RTS11. RTS11 cells were pre-treated with CgPACAP-38 (0.02 and 0.1 μM), 0.1 μM of hPACAP-38, or 0.1 μM of HSP70 peptide 24 h before the exposure to live *Y. ruckeri,* and the cfu/mL was calculated on day 3 post-infection (day 4 after PACAP treatment). Data represents the mean ± SD of three replicates. Differences among treatments were considered to be significantly different when compared to the No PACAP control (i.e., RTS11 exposed to only live *Y. ruckeri*, dashed line) by a one-way ANOVA followed by a Fisher’s least significant difference (LSD) post-hoc test. Different lowercase letters represent statistically significant differences at *p* < 0.05. The significance of the letters is indicated in Supplementary Table S4.

We next assessed the impact of PACAP on bacterial growth after infection with *Y. ruckeri* using the RTS11 cell line as an in vitro model. RTS11 cells were exposed to different PACAP concentrations 24 h prior to infection, and bacteria in the culture supernatant were quantified three days post-infection (day 4 after PACAP exposure). We observed that only the treatment with the higher dose of CgPACAP-38 tested (0.1 μM) was able to significantly decrease the bacterial count (cfu/mL) in the supernatant of RTS11 exposed to *Y. ruckeri* (*p* < 0.05) (Figure 6). There was not a significant impact of either a lower dose of CgPACAP-38, hPACAP-38, or an HSP70 control peptide on the number of viable bacteria in the cell culture supernatant (*p* > 0.05). 

### 2.5. Effect of PACAP-38 on Pro-Inflammatory Cytokines in RTS11 Infected with Yersinia ruckeri

**Figure 7:**
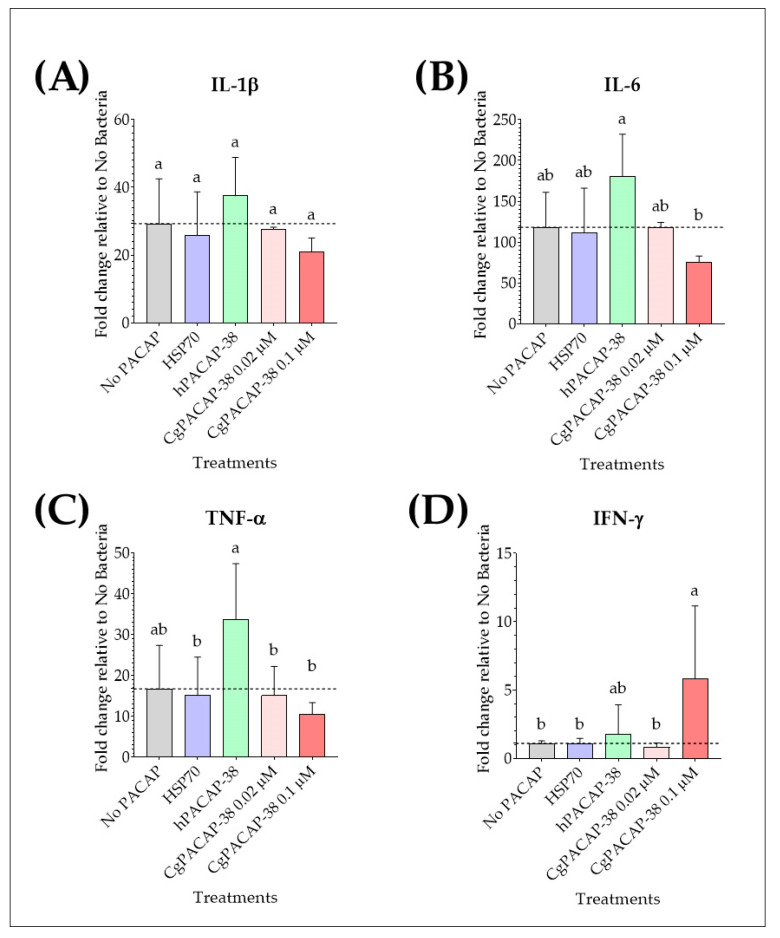
Relative expression levels of pro-inflammatory cytokines in RTS11 cells during live infection with *Yersinia ruckeri* (MOI of 0.001) after PACAP pre-treatment. RTS11 cells were pre-treated with CgPACAP-38 (0.02 and 0.1 μM), 0.1 μM of hPACAP-38, or 0.1 μM of HSP70 peptide 24 h before exposure to *Y. ruckeri,* and the relative expression levels of IL-1β (**A**), IL-6 (**B**), TNF-α (**C**), and IFN-γ (**D**) were analyzed on day 3 post-infection (day 4 after PACAP treatment). Relative expression was determined following the 2^−ΔΔCT^ method, and EF-1α was used as the reference gene. Data is expressed as fold change relative to the expression level in the No Bacteria group and was represented as the mean ± SD of 3 experimental replicates. Differences among treatments were considered to be significantly different when compared to the No PACAP control (RTS11 exposed to *Y. ruckeri*, dashed line) by a one-way ANOVA followed by a Fisher’s least significant difference (LSD) post-hoc test. Different lowercase letters represent statistically significant differences at *p* < 0.05. The significance of the letters is indicated in Supplementary Table S5.

We analyzed the effect of PACAP pre-treatment on pro-inflammatory cytokines IL-1β, IL-6, TNF-α, and IFN-γ in RTS11 cells after challenge with *Y. ruckeri* at day 3 post-infection (day 4 after PACAP exposure). *Y. ruckeri* infection *per se* caused an up-regulation of the expression of pro-inflammatory cytokines IL-1β, IL-6, and TNF-α (*p* < 0.01). For IL-1β expression, there were no significant differences among treatments post-infection when compared to the RTS11 cells exposed to a live pathogen alone (No PACAP control) (*p* > 0.05) (Figure 7A). Treatment with 0.1 μM hPACAP-38 was able to significantly increase the expression of IL-6 compared to 0.1 μM of CgPACAP-38 (*p* < 0.05) (Figure 7B), while TNF-α expression was significantly up-regulated by hPACAP-38 treatment compared to both doses of CgPACAP-38 (0.02 and 0.1 μM) (*p* < 0.05) and 0.1 μM of HSP70 peptide (*p* < 0.05) (Figure 7C). Interestingly, only the higher concentration of CgPACAP-38 assayed (0.1 μM) showed a significant increase in IFN-γ expression compared to the No PACAP control, 0.1 μM of HSP70 peptide, and 0.02 μM of CgPACAP-38 (*p* < 0.05, respectively) (Figure 7D). 

### 2.6. Effect of PACAP-38 on Anti-Inflammatory Cytokines, MYD88 Signal Transduction Adaptor Gene, and Antimicrobial Peptides in RTS11 Infected with Yersinia ruckeri

**Figure 8:**
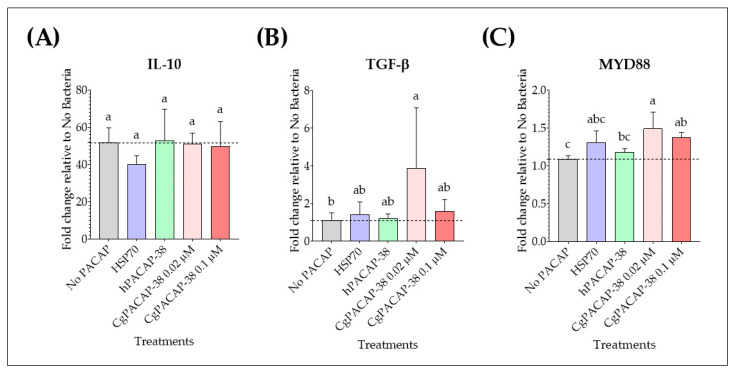
Relative expression levels of anti-inflammatory cytokines and immune signal transduction genes in RTS11 cells during live infection with *Yersinia ruckeri* (MOI of 0.001) after PACAP pre-treatment. RTS11 cells were pre-treated with CgPACAP-38 (0.02 and 0.1 μM), 0.1 μM of hPACAP-38, or 0.1 μM of HSP70 peptide 24 h before exposure to live *Y. ruckeri,* and the relative expression levels of IL-10 (**A**), TGF-β (**B**), and MYD88 (**C**) were analyzed on day 3 post-infection (day 4 after PACAP treatment). Relative expression was determined following the 2^−ΔΔCT^ method, and EF-1α was used as the reference gene. Data is expressed as fold change relative to the expression level in the No Bacteria group and was represented as the mean ± SD of 3 experimental replicates. Differences among treatments were considered to be significantly different when compared to the No PACAP control (RTS11 exposed to *Y. ruckeri*, dashed line) by a one-way ANOVA followed by a Fisher’s least significant difference (LSD) post-hoc test. Different lowercase letters represent statistically significant differences at *p* < 0.05. The significance of the letters is indicated in Supplementary Table S6.

We observed that at 3 days post-infection, *Y. ruckeri* infection caused an up-regulation of the expression of anti-inflammatory cytokines IL-10 and MYD88 (*p* < 0.01, respectively); however, there were no significant differences in the expression levels of the cytokine IL-10 among treatments when compared to the No PACAP control (*p* > 0.05) (Figure 8A). In contrast, a dose of 0.02 μM of CgPACAP-38 induced an up-regulation of the anti-inflammatory cytokine TGF-β compared to the No PACAP control and 0.1 μM of hPACAP-38 (*p* < 0.05, respectively) (Figure 8B). Also, the gene expression of the signal transduction adaptor MYD88 was significantly up-regulated with both doses of CgPACAP-38 (0.02 and 0.1 μM) compared to the No PACAP control (*p* < 0.01 and *p* < 0.05, respectively), while the lower dose of CgPACAP-38 significantly increased the expression of MYD88 compared to 0.1 μM of hPACAP-38 (*p* < 0.05) (Figure 8C).

Taking into account the impact of PACAP on the bacterial load after the in vitro live infection of the RTS11 cell line, we studied the expression levels of the antimicrobial peptides Cathelicidin-1 and Hepcidin. In spite of a significant decrease in the bacterial count in the supernatant of RTS11, we did not find any significant differences in the mRNA levels of both antimicrobial peptides (*p* > 0.05) (Supplementary Figure S1 and Supplementary Table S7). 

## 3. Discussion

The neuropeptide PACAP has been widely studied for its role in immunity and antimicrobial activity [31]. In fish, PACAP has been shown to have a physiological role in enhancing growth performance and food intake [32,33,34] and to also play a role as a regulator of the teleost fish immune system [6,32]. A new oiled feed formulation containing the peptide was developed and applied to rainbow trout fingerlings [35]. After two months of dietary supplementation with PACAP, we observed a marked improvement in the polyunsaturated fatty acid content in trout muscle, which provides various beneficial health effects. We also observed a significant change in the intestinal villi and a positive transcriptional modulation of different cytokines in the head kidney after PACAP oral delivery. However, research is still scarce, and more studies are needed to elucidate PACAP’s function as an antimicrobial agent. In this study, we first tested the idea that PACAP exerts a role in the immune response of rainbow trout, as its expression is stimulated in a natural infection by *Y. ruckeri*. Studies aimed at understanding the immune response against diseases in fish are important to generate new knowledge related to pathogenesis and immunity against infections. Also to develop more effective treatment and vaccination alternatives, which will contribute to the reduction of aquatic diseases and the impact they have, for instance, on rainbow trout farming.

Herein, after confirmation of the presence of ERM in naturally infected rainbow trout by the clinical signs of the disease, we observed a significant increase in PACAP-38, PRP/PACAP, and VPAC2 receptor transcriptional profiles in the spleen of Yersiniosis-symptomatic fish. The results suggest that PACAP splicing variants and the VPAC2 receptor play a role in the response of rainbow trout against natural infection by *Y. ruckeri*. Previously, Gorgoglione et al. [4] conducted an infection trial with *Y. ruckeri* in brown trout and demonstrated a significant increase in the transcription of PACAP and PRP/PACAP encoding genes in the spleen and kidney of infected fish compared to controls. Bacterial burden was also positively correlated with PACAP expression in both tissues. However, during ERM in brown trout, VPAC2 showed a large induction in the kidney with no significant modulation occurring in the spleen. The expression pattern of VPAC2 was also positively correlated with the bacterial burden in the kidney. The differences observed here could be related to species specificity, the bacteria strain used, and/or the fact that the study in brown trout was done by artificial infection with the bacteria. More studies involving natural *Y. ruckeri* infections in different salmonids will provide more insights into the role of the VIP-PACAP system in different immune-related tissues.

Previous research has demonstrated the direct antimicrobial activity of human PACAP-38 and variants [2] and of synthetic *C. gariepinus* PACAP-38 against gram-negative and gram-positive bacteria of biomedical and aquaculture importance [5,6]. In the present study, synthetic *C. gariepinus* PACAP-38 and human PACAP-38 were assessed for their direct antimicrobial activity against a 2018 isolate of the bacterial pathogen *Y. ruckeri*. Fish PACAP-38 had moderate direct antibacterial activity against *Y. ruckeri* when selective TSB was used. It was able to inhibit up to 60% of the bacterial growth only at the higher concentrations assayed (from 50 to 100 µM), while human PACAP was ineffective against *Y. ruckeri* in the same conditions. These results are different from those obtained by Lugo et al. [5] using LB media. These authors found 100% inhibition of bacteria growth using 50 μM of synthetic CgPACAP-38 against *Yersinia ruckeri* strain 4319/03, which suggests that PACAP direct antimicrobial activity depends on the media and strain used. Both CgPACAP-38 and hPACAP-38 had a significant enhancement of their direct antimicrobial activity against *Y. ruckeri* in the complete absence of NaCl salt in LB media. They inhibited bacterial growth by more than 90%, from 12.5 μM to the highest concentration of 50 μM. Previous studies demonstrated lag times and specific growth rates for bacteria with gradients of different pH, temperature, and salt concentrations [36,37,38], and those parameters can also affect the effectiveness of the antimicrobial compounds in a dose-dependent manner. For instance, the microbicidal activity of recombinant human intestinal defensin 5 (rHD-5) against *Salmonella typhimurium* was reduced in the presence of salt, and its activity was abolished at 100 mM NaCl. However, at all salt concentrations tested, rHD-5 remained bactericidal to *Listeria monocytogenes* [39]. 

We also evaluated for the first time the antimicrobial activity and immunomodulatory function of fish PACAP-38 within an in vitro live infection model consisting of the monocyte/macrophage-like rainbow trout cell line RTS11 infected with *Y. ruckeri*. Our results revealed that pre-treatment of RTS11 with 0.1 μM of CgPACAP 24 h before infection significantly reduced *Y. ruckeri* growth compared to non-treated cells. The results are consistent with the results of Semple et al. [6], who showed that pre-treatment with CgPACAP-38 at concentrations of 0.002, 0.02, and 0.1 μM significantly reduced the growth of *F. psychrophilum* in RTS11 at day 2 post-infection. They also observed that the two highest concentrations still significantly reduced bacterial growth at day 3 post-infection. 

In the present study, we observed that RTS11 cells responded to the bacterial exposure with an up-regulation of the mRNA expression levels of a 29-fold increase in IL-1β, a 118-fold increase in IL-6, and a 17-fold increase in TNF-α. However, the synthetic CgPACAP-38 had no effect on these cytokines expression at day 3 post-infection with *Y. ruckeri*, but it increased the IFN-γ response at this time point. RTS11 cells pre-treated with CgPACAP-38 and infected with *F. psychrophilum* produced a significant up-regulation of IL-1β at day 3 after infection, while IL-6 and TNF-α were up-regulated from day 1 post-infection [6]. A previous study revealed a dose/time-dependent modulation of gene expression by *Y. ruckeri* flagellin in RTS11 cells, where IL-1β, TNF- α, and IL-6 reached their highest levels at 1 h after stimulation with a dose of 100 ng/mL of the flagellin. No up-regulation of the expression of adaptive cytokines such as IFN-γ was found [40]. This suggests that the pro-inflammatory response in RTS11 may be influenced by the type of stimuli. 

CgPACAP-38 promoted up-regulation of the anti-inflammatory cytokine TGF-β and of the signal transduction adaptor MYD88. Macrophages and lymphocytes possess different receptors of the VIP/PACAP family [41]. Lower doses of IFN-γ in the effector Th2 cells in an asthma model were shown to have an anti-inflammatory function in vivo [42]. In teleost fishes, similar to mammals, there is a polarization of IFN-γ immune responses from the macrophage point of view, for which we can presumably obtain a balance between the ‘inflammatory’ M1 type of macrophages and the ‘healing’ M2 type of macrophages [43]. Endogenous factors, like the immunosuppressive cytokines IL-10 and TGF-β, are also actively involved in the regulation of the immune balance [44], while PACAP receptors can activate a broad range of signaling pathways [45]. Recently, we observed that synthetic CgPACAP-38 may exert an immunomodulatory effect in African catfish against the human pathogen *Pseudomonas aeruginosa*, presumably through a MyD88-dependent pathway in the spleen and head kidney [8]. Our findings in the current study support the idea that CgPACAP-38 could play a role in the modulation of the inflammatory reaction while the TLR signaling cascade remains activated. This can ultimately contribute to the maintenance of homeostasis during the effective eradication of the infection. 

The single exposure of RTS11 cells to CgPACAP-38 24 h prior to infection with *Y. ruckeri* did not produce an up-regulation of the antimicrobial peptides Cathelicidin-1 and Hepcidin in this model, and no correlation was observed with the bacterial growth inhibition. Nevertheless, we are only analyzing day 3 post-bacterial exposure. We do not know if there were changes in the gene expression of these or other antimicrobial peptides earlier. In vivo intraperitoneal administration of synthetic CgPACAP-38 to African catfish significantly induced the expression of pardaxin and hepcidin in the kidney and spleen 24 or 48 h after infection with *P. aeruginosa* [8]. Antimicrobial peptides are ‘first line’ defense mechanisms essential in the protection against pathogens in multicellular organisms [46]. The exposure of rainbow trout fry to a high dose of *Y. ruckeri* stimulated the expression of hepcidin in the first hours post-infection [47]. Here, the expected increased magnitude of the antimicrobial peptide responses was not observed; nevertheless, bacterial clearance in this live infection model may be due to other antimicrobial compounds like acute phase proteins, lysozymes, catalase, iNOS, and even other AMPs such as Defensin-1. 

We observed different results in the RTS11 in vitro model using CgPACAP-38 or hPACAP-38. These peptides share an 89% sequence identity at the amino acid level. The differences are in the C-terminal region, where 3 Lysine found in hPACAP-38 (positions 29, 32, and 36) is changed to arginine in CgPACAP-38 and 1 Valine in hPACAP-38 (position 35) is changed to phenylalanine in CgPACAP-38. The evolutionary pressure has acted to strongly preserve the primary sequence of the N-terminal domain (residues 1–27) of PACAP-38, which supports the importance of this region for its biological activity. There are two conserved regions at the N-terminal of PACAP-38 sequences (His1, Gly4, Phe6, and Asp8) and (Val19, Leu23, Val26, and Leu27) with predictive antimicrobial significance. Thus, the positively charged C-terminal PACAP (28–38) most likely has evolved and developed PACAP-species-specific functions [5,48]. This could explain the enhanced killing activity of CgPACAP-38 against a fish pathogen in this in vitro model compared to hPACAP-38.

In conclusion, this study demonstrated the effective antimicrobial and immunostimulatory properties of CgPACAP-38 against an important bacterial pathogen for rainbow trout. The results provided an insight into the immune response in fish affected by *Y. ruckeri* in a natural infection. This study also revealed PACAP-38’s potential properties to prevent and fight bacterial diseases in rainbow trout as an option to reduce the use of antibiotics in aquaculture and, thus, prevent the appearance of antimicrobial-resistant pathogens. Future research will be focused on the oral administration of synthetic CgPACAP-38 in the context of an oiled-based feed formulation and the evaluation of its effectiveness in the prevention of different important bacterial pathogens in rainbow trout aquaculture, such as *Flavobacterium psychrophilum*, *Yersinia ruckeri*, and *Aeromonas salmonicida*. 

## 4. Materials and Methods

### 4.1. Animals

For studying the effect of natural infection with *Y. ruckeri*, samples were taken from 67 rainbow trout with an approximate weight of 100–250 g. These trout came from semi-intensive fish farms in the Central Highlands of Peru (Jauja, Huaraz, Huancayo, Huaura, and Concepción provinces) that presented disease outbreaks suggestive of Yersiniosis during the period February–June 2018 and the summer–fall seasons. Temperature influences the development and severity of yersiniosis disease [14]. The optimal water temperature to produce rainbow trout oscillates between 11 and 16 °C; higher temperatures increase the risk of disease. Fish were separated into two groups; the first group consisted of 32 trout naturally infected by *Y. ruckeri*. The second group included 35 clinically healthy trout (without evidence of lesions or changes in behavior) from the same pools where disease outbreaks occurred. This research was approved by the Institutional Ethics Committee for the Use of Animals (CIEA) of the Universidad Peruana Cayetano Heredia, as indicated in the certificate 049-09-18.

### 4.2. Sample Collection from Naturally Infected Fish and Clinically Healthy Trout

Fish were euthanized with an overdose (100 mg/L) of tricaine methanesulfonate (MS-222, Sigma-Aldrich, St louis, MO, USA), followed by severing the spinal nerve to ensure mortality [49,50]. After an evaluation for the presence of external lesions, fish were cleaned with 70° ethanol before proceeding with the dissection according to the protocol described by Meyers [51]. Fish with clinical signs of disease were dissected, the abdominal wall was removed, and the internal organs were exposed and examined macroscopically for any gross abnormalities or internal lesions. To isolate the bacteria in these fish, samples were taken aseptically from the spleen and head kidney and cultured in tryptic soy agar (TSA). For all trout, tissue samples were obtained from the spleen, head, kidney, and skin and placed into 1.5 mL tubes containing 1 mL of RNA Later (Sigma). The tissue samples containing RNA were later stored at −70 °C until use. 

### 4.3. Identification of Y. ruckeri in Naturally Infected Fish and Clinically Healthy Trout 

Inoculated TSA plates were incubated at 25 °C for 24 h. Subsequently, the identification of the colonies was carried out considering their round shape, small size, bright cream color, and defined regular borders, followed by gram staining (negative bacilli) and catalase (+) and oxidase (−) tests. A presumptive colony was taken for each sampled fish; they were enriched in brain heart infusion (BHI) broth at 25 °C for 24 h, followed by centrifugation to obtain a bacterial pellet. Bacterial DNA was extracted using the commercial Wizard^®^ Genomic DNA Purification kit (Promega), according to the manufacturer’s instructions. For molecular confirmation of *Y. ruckeri* infection, the polymerase chain reaction (PCR) protocol established by Gibello et al. [52] for the 16S rRNA (GeneBank Accession Number EU401667) gene was followed. Each 20 μL PCR reaction included 1 μL of DNA, a concentration of 10 pmol for each primer, 2 mM of each deoxynucleoside triphosphate (dNTP), 10 μL of Taq polymerase buffer, and 5 U/μL of Taq polymerase. The primers used are shown in Table 1. The Mastercycler ™ Nexus thermal cycler (Eppendorf, Hamburg, Germany) was used under the following conditions: initial denaturation for 5 min at 92 °C, followed by 35 cycles for 1 min at 92 °C, annealing at 60 °C for 1 min, extension at 72 °C for 1 min, and final extension at 72 °C for 5 min. A positive control of *Y. ruckeri* ATTC^®^ 29473 and a negative control composed of DNase-free water were included. Electrophoresis at 80 V/cm for 90 min in 1.0% agarose gel (Calbiochem, San Diego, CA, USA) using buffer TAE 1X was carried out in the Enduro™ Gel Systems and Power Supplies equipment (Labnet International, Edison NJ, USA), including the 100 bp DNA Ladder (Promega, Madison, WI, USA). The gel was dyed with 0.5 μg/mL of ethidium bromide (Merck Millipore, Burlington, MA, USA) for 20 min, and bands were observed in the MiniBIS transilluminator (DNR Bio-Imaging Systems, USA).

### 4.4. RNA Extraction and cDNA Synthesis 

Samples of RNA later stored in dry ice (−70 °C) were sent to Dr. Brian Dixon’s Laboratory at the Department of Biology, University of Waterloo, Canada, for the following procedures: Ten micrograms of tissue samples (spleen, head, kidney, and skin) were transferred to 5 mL eppendorf tubes containing 1 mL of TRIzol^TM^ Reagent (Invitrogen, Waltham, MA, USA), and the manufacturer’s protocol was followed. RNA samples were quantified using the Take3 plate of a Synergy H1 plate reader (BioTek Instruments, Winooski, VT, USA). For the next step, 5 μg of RNA were treated with 10 units of DNase I (Thermo Fisher Scientific, Waltham, MA, USA) to remove any genomic DNA that could be contaminating the sample at 25 °C for 30 min. The enzyme was then removed using a column from an RNA purification kit (Norgen Biotek, Thorold, ON, Canada). RNA was quantified again and stored at −80 °C. Five hundred nanograms of total RNA were used to synthesize cDNA with a qScript cDNA Supermix (Quanta Biosciences, Rockville, MD, USA) following the manufacturer’s instructions. Synthesized cDNA samples were maintained at −80 °C until further use. 

### 4.5. Gene Expression by qRT-PCR 

The relative expression of *Y. ruckeri* 16S rRNA, PACAP splicing variants, and VPAC2 receptor genes in *Y. ruckeri* naturally infected fish and healthy fish was assessed by qRT-PCR. Each 10 μL qRT-PCR reaction included 2.5 μL of cDNA (25 ng/μL diluted 1:10 in RNase-free water for cytokine genes and diluted 1:5 in RNase-free water for PACAP splicing variants and receptor genes), 2x SYBR^®^ Green qPCR Master Mix (Wisent Bioproducts, Saint-Jean-Baptiste, QC, Canada), and the correspondent forward and reverse primers (Sigma-Aldrich, St louis, MO, USA) for a final concentration of 0.25 μM. Primers used are shown in Table 1. Reactions were run on the LightCycler^®^ 480 II System (Roche, Basel, Switzerland). For each plate, all samples were run in triplicate, and a calibrator and a non-template control were included. The program consisted of pre-incubation for 2 min at 95 °C, 40 cycles of denaturation for 10 s at 95 °C, annealing for 5 s at 60 °C, and finally an extension for 8 s at 72 °C. A melting curve for each plate was used to verify the amplification of a unique product; this was obtained by reading the fluorescence at each grade between 65 and 97 °C every 5 s. Elongation factor-1 alpha (EF-1α) was used as the reference gene. Each gene expression was normalized to the expression of the reference gene, and the levels of expression were analyzed using the 2^−ΔΔCT^ method [57]. Data was expressed as fold change relative to the expression level in the healthy trout (mean ± SD, N = a minimum of 21 infected and a minimum of 17 healthy trout). 

### 4.6. In Vitro Trials to Determine the Activity of PACAP as an Antimicrobial and Immunostimulant Agent

#### 4.6.1. Peptide Synthesis and Bacterial Strain

*Clarias gariepinus* synthetic PACAP-38 (amino acid sequence of HSDGIFTDSYSRYRKQMAVKKYLAAVLGRRYRQRFRNK, MW of 4.7 kDa) and human synthetic PACAP-38 (amino acid sequence of HSDGIFTDSYSRYRKQMAVKKYLAAVLGKRYKQRVKNK, MW of 4.5 kDa) were purchased from (Bio Basic, Markham, ON, Canada) with a minimum of 95% purity. A synthetic peptide fragment of rainbow trout HSP70 (amino acid sequence of CGDQARTSSGASSQ, MW of 1.3 kDa) was purchased from Biomatik, Kitchener, ON, Canada with 98% purity. 

The *Y. ruckeri* strain used for the in vitro experiments was isolated in West Virginia in 2018 and donated by Dr. Niels C. Bols from the Department of Biology, University of Waterloo, Canada. Bacteria were cultured from a glycerol stock into TSA and grown for 24 h at 25 °C. After being checked for purity, 15–20 colonies were obtained to inoculate 4 mL of TSB and grown for 24 h at 25 °C. After the incubation period, the OD_600nm_ of the bacterial growth was measured. This procedure was repeated five times in the same conditions to get a consistent OD value that can be trusted to estimate the multiplicity of infection (MOI). For every bacterial culture, a standard plate count (SPC) was performed to confirm the anticipated cfu/mL.

#### 4.6.2. Direct Antibacterial Activity

An in vitro antimicrobial activity assay was performed by a broth microdilution peptide method described by Otvos and Cudic [58], with minor modifications. Briefly, single colonies from previously grown *Y. ruckeri* in TSA plates at 25 °C for 24 h were inoculated in 3 mL of TSB and were grown at 25 °C for 16 h. After incubation, 1 mL of bacterial culture was centrifuged, and the pellet was re-suspended in 4 mL of fresh media to get an OD_600nm_ of 0.1–0.4. Then, the bacterial suspension was diluted in TSB to obtain an OD_600nm_ of 0.001, which is approximately equivalent to 1 × 10^5^ cfu/mL. The broth microdilution peptide assay was performed using a flat-bottom 96-well plate (Thermo Fisher Scientific). All wells were filled with 90 μL of bacterial suspension and 10 μL of CgPACAP-38 or hPACAP-38 at different concentrations from 2.5 to 100 μM (diluted in sterile PBS (Gibco, Billings, MT, USA). Positive control wells consist of 10 μL of PBS instead of PACAP with 90 μL of bacterial suspension, and negative control wells contain 10 μL of PBS and 90 μL of TSB. All samples were tested in triplicate. After 24 h of incubation at 14 or 25 °C, the absorbance was measured at 600 nm using a microplate reader (BioTek). The growth inhibition curves were generated by plotting the OD at 600 nm and the peptide concentration. The MIC was considered to be the lowest concentration of PACAP at which no bacterial growth was detected (an OD_600nm_ of 0). The percentage of inhibition of the bacterial growth was determined by the formula:

(1) $$\%\;of\;inhibition=100-\left\{\left[\frac{ODsample-ODblank}{OD+control-ODblank}\right]*\;100\right\}$$

For antibacterial activity with different NaCl concentrations in LB media, the recipe was made from individual components (1% tryptone, 0.5% yeast extract, and 0, 0.5, or 1% sodium chloride (NaCl)). The broth microdilution peptide assay was performed as described above, where all wells were filled with 90 μL of bacterial suspension and 10 μL of CgPACAP-38 or hPACAP-38 (0, 2.5, 12.5, 25, and 50 μM diluted in sterile PBS), and the plates were incubated for 24 h at 14 °C. 

#### 4.6.3. Pre-Treatment of RTS11 with PACAP Followed by Infection with Live *Y. ruckeri*

The rainbow trout monocyte/macrophage-like cell line, RTS11, was maintained as described previously by Sever et al. [59]. In 6-well tissue culture plates (ThermoFisher, Waltham, MA, USA), RTS11 cells were seeded at a density of 1.5 × 10^6^ cells/well (in 1.5 mL of L-15 media (Gibco)) with no antibiotics and maintained for 16 h at 25 °C. Cells were exposed to 0.02 μM or 0.1 μM of CgPACAP-38, 0.1 μM of hPACAP-38, 0.1 μM of HSP70 peptide, or a no PACAP control in a final volume of 4 mL per well. Following this single exposure to the treatments, all experimental plates were returned to the incubator and maintained for 24 h at 25 °C. On the next day, 0.1 mL of the correspondent diluted bacterial suspension (in L-15) of *Y. ruckeri* was added to each well to expose the cells with a MOI of 0.001 (equivalent to 1.5 × 10^6^ cfu/mL). Control wells with only L-15 media that were not infected were included as well. All samples were tested in triplicate. The plates were incubated at 25 °C. On day three post-infection (day four after PACAP exposure), in order to compare bacterial growth among treatments, 100 μL of each cell culture supernatants were seeded in TSA plates and placed for 24 h at 25 °C before standard plate counting (in triplicate plates) to determine the number of viable bacterial cells in the supernatant. The remaining supernatant was collected from experimental wells, and adherent cells were mechanically dislodged using a sterile 23 cm cell scraper (ThermoFisher, Waltham, MA, USA) and added to the supernatant of the respective wells. All wells were then washed with 1 mL of PBS, which was also added to the appropriate supernatant/cell mixture. The cells were centrifuged at 4 °C for 5 min at 500× *g*, washed once with 5 mL of PBS, and the resulting cell pellets after centrifugation were stored at −80 °C for future RNA extraction.

#### 4.6.4. RNA Extraction and cDNA Synthesis

RNA was extracted from RTS11 cell pellets (1.5 × 10^6^ cells) using an RNeasy RNA Extraction Kit (Qiagen, Germantown, MD, USA) as described by the manufacturer. To remove any contaminating genomic DNA, all RNA samples were treated with DNase I (Thermo Scientific). RNA samples were quantified as described above, and cDNA was synthesized from 500 ng of total RNA using the qScript cDNA Supermix (Quanta Biosciences, Rockville, MD, USA) in accordance with the manufacturer’s instructions. For a no-template control, 500 ng of RNA suspended in 20 μL of DEPC water was included in the cDNA synthesis reaction without reverse transcriptase.

#### 4.6.5. Gene Expression by qRT-PCR

To assess transcript levels of IL-1β, IL-6, TNF-α, IFN-γ, IL-10, TGF-β, MYD88, Cathelicidin-1, and Hepcidin in RTS11 cells, qRT-PCR analysis was completed as described before. All PCR reactions contained 2.5 μL of cDNA (25 ng/μL diluted 1:10 in RNase-free water), 2x WISENT ADVANCED^TM^ qPCR Master Mix (Wisent), and forward and reverse primers (Sigma Aldrich) to a final working concentration of 0.25 μM. The sequences for all primer sets are outlined in Table 1. Each gene expression was normalized to the expression of the reference gene EF-1α, and the levels of expression were analyzed using the 2^−ΔΔCT^ method [57]. Data was expressed as fold change relative to the expression level in the No Bacteria group and was represented as the mean ± SD of 3 experimental replicates.

### 4.7. Data Analysis 

Data handling and graphic representation were performed using Office Excel 2010 (Microsoft Corporation, Redmond, WA, USA) and GraphPad Prism version 8.0 for Windows (GraphPad Software, Boston, MA USA, www.graphpad.com, accessed on 19 January 2023), respectively. All statistical analyses were performed in GraphPad Prism version 8.0. The normality of the data was verified by the D’Agostino and Pearson bus normality tests. The homogeneity of variances was verified by Bartlett’s test. The statistical analysis was performed using an unpaired, two-tailed Student’s *t*-test to determine statistically significant differences in gene expression between infected and healthy trout. When variances were significantly different, a Mann–Whitney U test was performed. A two-way ANOVA followed by a Tukey’s multiple comparisons post-hoc test was performed to determine the significant differences in bacterial growth inhibition among LB salt concentrations. A one-way ANOVA followed by a Fisher’s least significant difference (LSD) post-hoc test was performed to determine in RTS11 cells the significant differences among treatments in bacterial growth and the gene expression analysis. P values less than 0.05 were considered statistically significant. The data was shown as mean ± standard deviation.

## Figures and Tables

**Table 1:** List of primers used in this study.

Gene	Primer Sequence (5′ to 3′) *	Reference
*Y. ruckeri* 16S rRNA	F: GCGAGGAGGAAGGGTTAAGTG R: GAAGGCACCAAGGCATCTCTG	Gibello et al. [52]
PACAP	F: AAATTGCTATAAGAAGTCCCCCATC R: GTATTTCTTGACTGCCATTTGCTTT	Lugo et al. [53]
PRP/PACAP	F: CTGGGTCAGTTATCAGCAAGAAAT R: TGTCTATACCTTTTCCCAAGGACTG	Lugo et al. [53]
VPAC2	F: CTCACTGTGACACGACAGTGATTCC R: GCTTTTCAGTTTCACCTCAACTTGT	Lugo et al. [53]
IL-1β	F: CCACAAAGTGCATTTGAAC R: GCAACCTCCTCTAGGTGC	Semple et al. [6]
IL-6	F: CTTCTACACGCTATCTCTCACTC R: CGTCTGTCCCGAGCT	Semple et al. [6]
TNF-α	F: GTGCAAAAGATACCCACC R: CACTGCACGGTGTCAG	Semple et al. [6]
IFN-γ	F: GAAGGCTCTGTCCGAGTTCA R: TGTGTGATTTGAGCCTCTGG	Chaves-Pozo et al. [54]
IL-10	F: GCCTTCTCCACCATCAGAGAC R: GATGCTGTCCATAGCGTGAC	Inoue et al. [55]
TGF-β	F: TGTGGGGAGACAACACAAGG R: AAACCAGCGCCATCAAAAAGG	This study
MYD88	F: GACAAAGTTTGCCCTCAGTCTCT R: CCGTCAGGAACCTCAGGATACT	This study
Cathelicidin-1	F: ATGGGAAACTAATGATGTGC R: CGGTCAGTGTTGAGGGTATT	Broekman et al. [56]
Hepcidin	F: GCTTCTGCTGCAAATTCTGAGG R: GTACAAGACTGAGGTTGTGCAG	This study
EF-1α	F: CGCACAGTAACACCGAAACTAATTAAGC R: GCCTCCGCACTTGTAGATCAGATG	Semple et al. [6]

* Direction of the primers is presented as F and R representing forward and reverse primers, respectively.

## Data Availability

All relevant data are within the manuscript and its Supporting Information Files.

## Supplementary Materials

The following supporting information can be downloaded at: https://www.mdpi.com/article/10.3390/antibiotics12101484/s1, Figure S1: Relative expression levels of antimicrobial peptides in RTS11 cells during live infection with *Yersinia ruckeri* (MOI of 0.001) after PACAP pre-treatment. RTS11 cells were pre-treated with CgPACAP-38 (0.02 and 0.1 μM), 0.1 μM of hPACAP-38 or 0.1 μM of HSP70 peptide 24 h before the exposure to live *Y. ruckeri* and the relative expression levels of Cathelicidin-1 (**A**) and Hepcidin (**B**) were analyzed on day 3 post-infection (day 4 after PACAP treatment). Relative expression was determined following the 2^−ΔΔCT^ method and EF-1α was used as the reference gene. Data was expressed as fold change relative to the expression level in the No Bacteria group, and was represented as the mean ± SD of 3 experimental replicates. Differences among treatments were considered to be significantly different when compared to the No PACAP control (RTS11 exposed to *Y. ruckeri,* dashed line) by a One-way ANOVA followed by a Fisher’s least significant difference (LSD) post-hoc test. Similar lowercase letters represents no statistically significant differences at *p* < 0.05; Supplementary Table S1: Molecular identification of *Yersinia ruckeri* 16S rRNA gene in rainbow trout (*Oncorhynchus mykiss*) by qRT-PCR; Supplementary Table S2. Statistical analyzed performed to the direct antimicrobial activity of synthetic *Clarias gariepinus* PACAP-38 against a *Y. ruckeri* isolate by broth microdilution peptide assay in Luria-Bertani broth; Supplementary Table S3. Statistical analyzed performed to the direct antimicrobial activity of synthetic human PACAP-38 against a *Y. ruckeri* isolate by broth microdilution peptide assay in Luria-Bertani broth; Supplementary Table S4. Statistical analyzed performed to the quantification of *Y. ruckeri* by standard plate count (SPC) of cell culture media during live infection (MOI of 0.001) of PACAP-treated RTS11; Supplementary Table S5. Statistical analyzed performed to the relative expression levels of pro-inflammatory cytokines in RTS11 cells during live infection with *Y. ruckeri* (MOI of 0.001) after PACAP pre-treatment; Supplementary Table S6. Statistical analyzed performed to the relative expression levels of anti-inflammatory cytokines and MYD88 signal transduction adaptor gene in RTS11 cells during live infection with *Y. ruckeri* (MOI of 0.001) after PACAP pre-treatment; Supplementary Table S7. Statistical analyzed performed to the relative expression levels of antimicrobial peptides in RTS11 cells during live infection with *Y. ruckeri* (MOI of 0.001) after PACAP pre-treatment.
