# Supplementary material for: New Evidence for the Role of Pituitary Adenylate Cyclase-Activating Polypeptide as an Antimicrobial Peptide in Teleost Fish
Source: Antibiotics (Basel). 2023 Sep 27;12(10):1484. doi: 10.3390/antibiotics12101484 (PMC10604671; doi:10.3390/antibiotics12101484)
Supplement: Supplementary file 1 [file antibiotics-12-01484-s001.zip › antibiotics-2612427-supplementary.pdf]

antibiotics MDPI

Article

New Evidence for the Role of PACAP as an Antimicrobial Peptide in Teleost Fish

Janet Velázquez [1], Tania Rodríguez-Cornejo [2], Tania Rodríguez-Ramos [2], Geysi Pérez-Rodríguez [1], Laura Rivera [2], James Hugh Campbell [2], Lowia Al-Hussinee [2], Yamila Carpio [1,*], Mario Pablo Estrada [1,*] and Brian Dixon [2,*]

[1] Veterinary Immunology Project, Animal Biotechnology Department, Center for Genetic Engineering and Biotechnology (CIGB), P.O. Box 6162, Havana 10600, Cuba; janet.velazquez@cigb.edu.cu (J.V.); gey9412@gmail.com (G.P.-R.)

[2] Department of Biology, University of Waterloo, 200 University Ave W., Waterloo, ON, N2L3G1, Canada; tania.rodriguez@upch.pe (T.R.-C.); tania.rodriguez-ramos@uwaterloo.ca (T.R.-R.); l2rivera@uwaterloo.ca (L.R.); jhcampbell@uwaterloo.ca (J.C.); jlowia@gmail.com (L.A.-H.)

* Correspondence: yamila.carpio@cigb.edu.cu (Y. C.), mario.pablo@cigb.edu.cu (M. P. E.) and bdixon@uwaterloo.ca (B. D.)

Supplementary Table S1. Molecular identification of *Yersinia ruckeri* 16S rRNA gene in rainbow trout (*Oncorhynchus mykiss*) by qRT-PCR

Control Group		Infected Group	
**Sample**	**Mean of CT value**	**Sample**	**Mean of CT value**
**Control 3**	31.46	**Infected 1**	13.68
**Control 4**	32.91	**Infected 2**	14.24
**Control 5**	32.87	**Infected 3**	13.40
**Control 6**	31.35	**Infected 4**	14.39
**Control 7**	33.03	**Infected 5**	12.30
**Control 8**	30.60	**Infected 6**	13.61
**Control 9**	30.99	**Infected 7**	13.33
**Control 10**	33.28	**Infected 8**	15.47
**Control 11**	31.69	**Infected 9**	15.27
**Control 12**	31.61	**Infected 10**	13.68
**Control 13**	32.13	**Infected 11**	17.52
**Control 15**	33.66	**Infected 12**	13.49
**Control 16**	31.06	**Infected 13**	13.69
**Control 17**	32.28	**Infected 14**	13.39
**Control 18**	33.32	**Infected 15**	15.56
**Control 19**	32.40	**Infected 16**	14.85
**Control 20**	31.38	**Infected 17**	20.57
**Control 21**	32.58	**Infected 18**	15.30
**Control 22**	32.91	**Infected 19**	15.44
**Control 23**	32.24	**Infected 20**	13.17
**Control 24**	31.33	**Infected 21**	18.51
**Control 25**	31.68	**Infected 22**	17.46
**Control 26**	34.84	**Infected 23**	23.98
**Control 27**	35.13	**Infected 24**	21.56
**Control 34**	32.68	**Infected 25**	16.86
**Control 35**	30.98	**Infected 26**	20.55
**Control 37**	30.72	**Infected 27**	28.12
		**Infected 28**	13.15
		**Infected 29**	15.11
		**Infected 30**	19.68
		**Infected 31**	16.87
		**Infected 32**	21.77
**Average**	**32.26**	**Average**	**16.44**
**SD**	**1.16**	**SD**	**3.70**

Supplementary Table S2. Statistical analyzed performed to the direct antimicrobial activity of synthetic *Clarias gariepinus* PACAP-38 against a *Y. ruckeri* isolate by broth microdilution peptide assay in Luria-Bertani broth

Tukey's multiple comparisons test	Summary	Adjusted P Value
**0 µM CgPACAP-38**		
NaCl 1% vs. NaCl 0.5%	ns	>0.9999
NaCl 1% vs. NaCl 0%	ns	>0.9999
NaCl 0.5% vs. NaCl 0%	ns	>0.9999
**2.5 µM CgPACAP-38**		
NaCl 1% vs. NaCl 0.5%	*	0.0358
NaCl 1% vs. NaCl 0%	***	0.0007
NaCl 0.5% vs. NaCl 0%	ns	0.1502
**12.5 µM CgPACAP-38**		
NaCl 1% vs. NaCl 0.5%	**	0.0038
NaCl 1% vs. NaCl 0%	****	<0.0001
NaCl 0.5% vs. NaCl 0%	****	<0.0001
**25 µM CgPACAP-38**		
NaCl 1% vs. NaCl 0.5%	****	<0.0001
NaCl 1% vs. NaCl 0%	****	<0.0001
NaCl 0.5% vs. NaCl 0%	ns	0.1956
**50 µM CgPACAP-38**		
NaCl 1% vs. NaCl 0.5%	***	0.0003
NaCl 1% vs. NaCl 0%	***	0.0001
NaCl 0.5% vs. NaCl 0%	ns	0.8931

Supplementary Table S3. Statistical analyzed performed to the direct antimicrobial activity of synthetic human PACAP-38 against a *Y. ruckeri* isolate by broth microdilution peptide assay in Luria-Bertani broth

Tukey's multiple comparisons test	Summary	Adjusted P Value
**0 µM hPACAP-38**		
NaCl 1% vs. NaCl 0.5%	ns	>0.9999
NaCl 1% vs. NaCl 0%	ns	>0.9999
NaCl 0.5% vs. NaCl 0%	ns	>0.9999
**2.5 µM hPACAP-38**		
NaCl 1% vs. NaCl 0.5%	ns	0.0569
NaCl 1% vs. NaCl 0%	****	<0.0001
NaCl 0.5% vs. NaCl 0%	****	<0.0001
**12.5 µM hPACAP-38**		
NaCl 1% vs. NaCl 0.5%	****	<0.0001
NaCl 1% vs. NaCl 0%	****	<0.0001
NaCl 0.5% vs. NaCl 0%	****	<0.0001
**25 µM hPACAP-38**		
NaCl 1% vs. NaCl 0.5%	****	<0.0001
NaCl 1% vs. NaCl 0%	****	<0.0001
NaCl 0.5% vs. NaCl 0%	****	<0.0001
**50 µM hPACAP-38**		
NaCl 1% vs. NaCl 0.5%	****	<0.0001
NaCl 1% vs. NaCl 0%	****	<0.0001
NaCl 0.5% vs. NaCl 0%	***	0.0009

Supplementary Table S4. Statistical analyzed performed to the quantification of *Y. ruckeri* by standard plate count (SPC) of cell culture media during live infection (MOI of 0.001) of PACAP-treated RTS11

Uncorrected Fisher's LSD	Summary	Individual P Value
No PACAP vs. hPACAP-38	ns	0.1049
No PACAP vs. HSP70	ns	0.4080
No PACAP vs. CgPACAP-38 0.02 μM	ns	0.0848
No PACAP vs. CgPACAP-38 0.1 μM	*	0.0237
hPACAP-38 vs. HSP70	ns	0.3743
hPACAP-38 vs. CgPACAP-38 0.02 μM	ns	0.8967
hPACAP-38 vs. CgPACAP-38 0.1 μM	ns	0.2979
HSP70 vs. CgPACAP-38 0.02 μM	ns	0.3132
HSP70 vs. CgPACAP-38 0.1 μM	ns	0.0842
CgPACAP-38 0.02 μM vs. CgPACAP-38 0.1 μM	ns	0.3502

Supplementary Table S5. Statistical analyzed performed to the relative expression levels of pro-inflammatory cytokines in RTS11 cells during live infection with *Y. ruckeri* (MOI of 0.001) after PACAP pre-treatment

Uncorrected Fisher's LSD	Summary	Individual P Value
**IL-1β**		
No PACAP vs. hPACAP-38	ns	0.3569
No PACAP vs. HSP70	ns	0.6856
No PACAP vs. CgPACAP-38 0.02 μM	ns	0.8416
No PACAP vs. CgPACAP-38 0.1 μM	ns	0.3987
hPACAP-38 vs. HSP70	ns	0.1982
hPACAP-38 vs. CgPACAP-38 0.02 μM	ns	0.2695
hPACAP-38 vs. CgPACAP-38 0.1 μM	ns	0.1132
HSP70 vs. CgPACAP-38 0.02 μM	ns	0.8365
HSP70 vs. CgPACAP-38 0.1 μM	ns	0.6210
CgPACAP-38 0.02 μM vs. CgPACAP-38 0.1 μM	ns	0.5004
**IL-6**		
No PACAP vs. hPACAP-38	ns	0.0952
No PACAP vs. HSP70	ns	0.8464
No PACAP vs. CgPACAP-38 0.02 μM	ns	0.9911
No PACAP vs. CgPACAP-38 0.1 μM	ns	0.2870
hPACAP-38 vs. HSP70	ns	0.0691
hPACAP-38 vs. CgPACAP-38 0.02 μM	ns	0.0935
hPACAP-38 vs. CgPACAP-38 0.1 μM	*	0.0207
HSP70 vs. CgPACAP-38 0.02 μM	ns	0.8551
HSP70 vs. CgPACAP-38 0.1 μM	ns	0.3653
CgPACAP-38 0.02 μM vs. CgPACAP-38 0.1 μM	ns	0.2911
**TNF-*α***		
No PACAP vs. hPACAP-38	ns	0.0653
No PACAP vs. HSP70	ns	0.8431
No PACAP vs. CgPACAP-38 0.02 μM	ns	0.8428
No PACAP vs. CgPACAP-38 0.1 μM	ns	0.5128
hPACAP-38 vs. HSP70	*	0.0468
hPACAP-38 vs. CgPACAP-38 0.02 μM	*	0.0468
hPACAP-38 vs. CgPACAP-38 0.1 μM	*	0.0308
HSP70 vs. CgPACAP-38 0.02 μM	ns	0.9997
HSP70 vs. CgPACAP-38 0.1 μM	ns	0.6297
CgPACAP-38 0.02 μM vs. CgPACAP-38 0.1 μM	ns	0.6299

**IFN-γ**		
No PACAP vs. hPACAP-38	ns	0.6968
No PACAP vs. HSP70	ns	0.9985
No PACAP vs. CgPACAP-38 0.02 μM	ns	0.8738
No PACAP vs. CgPACAP-38 0.1 μM	*	0.0316
hPACAP-38 vs. HSP70	ns	0.6954
hPACAP-38 vs. CgPACAP-38 0.02 μM	ns	0.5854
hPACAP-38 vs. CgPACAP-38 0.1 μM	ns	0.0569
HSP70 vs. CgPACAP-38 0.02 μM	ns	0.8754
HSP70 vs. CgPACAP-38 0.1 μM	*	0.0315
CgPACAP-38 0.02 μM vs. CgPACAP-38 0.1 μM	*	0.0248

Supplementary Table S6. Statistical analyzed performed to the relative expression levels of anti-inflammatory cytokines and MYD88 signal transduction adaptor gene in RTS11 cells during live infection with *Y. ruckeri* (MOI of 0.001) after PACAP pre-treatment

Uncorrected Fisher's LSD	Summary	Individual P Value
**IL-10**		
No PACAP vs. hPACAP-38	ns	0.8981
No PACAP vs. HSP70	ns	0.2110
No PACAP vs. CgPACAP-38 0.02 μM	ns	0.9434
No PACAP vs. CgPACAP-38 0.1 μM	ns	0.8494
hPACAP-38 vs. HSP70	ns	0.1734
hPACAP-38 vs. CgPACAP-38 0.02 μM	ns	0.8423
hPACAP-38 vs. CgPACAP-38 0.1 μM	ns	0.7612
HSP70 vs. CgPACAP-38 0.02 μM	ns	0.2347
HSP70 vs. CgPACAP-38 0.1 μM	ns	0.3393
CgPACAP-38 0.02 μM vs. CgPACAP-38 0.1 μM	ns	0.8993
**TGF-β**		
No PACAP vs. hPACAP-38	ns	0.9370
No PACAP vs. HSP70	ns	0.8007
No PACAP vs. CgPACAP-38 0.02 μM	*	0.0487
No PACAP vs. CgPACAP-38 0.1 μM	ns	0.6920
hPACAP-38 vs. HSP70	ns	0.8621
hPACAP-38 vs. CgPACAP-38 0.02 μM	ns	0.0558
hPACAP-38 vs. CgPACAP-38 0.1 μM	ns	0.7506
HSP70 vs. CgPACAP-38 0.02 μM	ns	0.0753
HSP70 vs. CgPACAP-38 0.1 μM	ns	0.8849
CgPACAP-38 0.02 μM vs. CgPACAP-38 0.1 μM	ns	0.0962
**MYD88**		
No PACAP vs. hPACAP-38	ns	0.4056
No PACAP vs. HSP70	ns	0.0664
No PACAP vs. CgPACAP-38 0.02 μM	**	0.0044
No PACAP vs. CgPACAP-38 0.1 μM	*	0.0420
hPACAP-38 vs. HSP70	ns	0.2552
hPACAP-38 vs. CgPACAP-38 0.02 μM	*	0.0177
hPACAP-38 vs. CgPACAP-38 0.1 μM	ns	0.1466
HSP70 vs. CgPACAP-38 0.02 μM	ns	0.1267
HSP70 vs. CgPACAP-38 0.1 μM	ns	0.6279
CgPACAP-38 0.02 μM vs. CgPACAP-38 0.1 μM	ns	0.3419

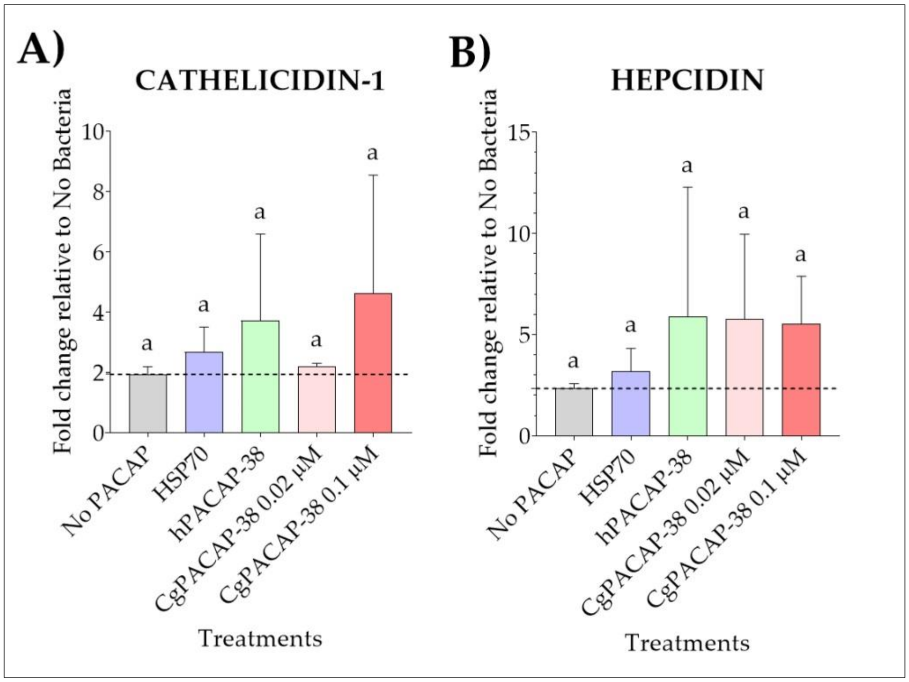

Supplementary Figure S1. Relative expression levels of antimicrobial peptides in RTS11 cells during live infection with *Yersinia ruckeri* (MOI of 0.001) after PACAP pre-treatment. RTS11 cells were pre-treated with CgPACAP-38 (0.02 and 0.1 μM), 0.1 μM of hPACAP-38 or 0.1 μM of HSP70 peptide 24 h before the exposure to live Y. ruckeri and the relative expression levels of Cathelicidin-1 **(A)** and Hepcidin **(B)** were analyzed on day 3 post-infection (day 4 after PACAP treatment). Relative expression was determined following the $2^{-\Delta\Delta CT}$ method and EF-1α was used as the reference gene. Data was expressed as fold change relative to the expression level in the No Bacteria group, and was represented as the mean ± SD of 3 experimental replicates. Differences among treatments were considered to be significantly different when compared to the No PACAP control (RTS11 exposed to *Y. ruckeri,* dashed line) by a One-way ANOVA followed by a Fisher's least significant difference (LSD) post-hoc test. Similar lowercase letters represents no statistically significant differences at $p < 0.05$.

Supplementary Table S7. Statistical analyzed performed to the relative expression levels of antimicrobial peptides in RTS11 cells during live infection with *Y. ruckeri* (MOI of 0.001) after PACAP pre-treatment

Uncorrected Fisher's LSD	Summary	Individual P Value
**Cathelicidin-1**		
No PACAP vs. hPACAP-38	ns	0.3471
No PACAP vs. HSP70	ns	0.6916
No PACAP vs. CgPACAP-38 0.02 μM	ns	0.8840
No PACAP vs. CgPACAP-38 0.1 μM	ns	0.1658
hPACAP-38 vs. HSP70	ns	0.5758
hPACAP-38 vs. CgPACAP-38 0.02 μM	ns	0.4221
hPACAP-38 vs. CgPACAP-38 0.1 μM	ns	0.6224
HSP70 vs. CgPACAP-38 0.02 μM	ns	0.8011
HSP70 vs. CgPACAP-38 0.1 μM	ns	0.3028
CgPACAP-38 0.02 μM vs. CgPACAP-38 0.1 μM	ns	0.2083
**Hepcidin**		
No PACAP vs. hPACAP-38	ns	0.2717
No PACAP vs. HSP70	ns	0.7830
No PACAP vs. CgPACAP-38 0.02 μM	ns	0.2892

No PACAP vs. CgPACAP-38 0.1 μM	ns	0.3741
hPACAP-38 vs. HSP70	ns	0.3980
hPACAP-38 vs. CgPACAP-38 0.02 μM	ns	0.9652
hPACAP-38 vs. CgPACAP-38 0.1 μM	ns	0.9131
HSP70 vs. CgPACAP-38 0.02 μM	ns	0.4214
HSP70 vs. CgPACAP-38 0.1 μM	ns	0.5127
CgPACAP-38 0.02 μM vs. CgPACAP-38 0.1 μM	ns	0.9442
